# Supplementary material for: The role of prefrontal cortex in the control of feature attention in area V4
Source: Nat Commun. 2019 Dec 16;10:5727. doi: 10.1038/s41467-019-13761-7 (PMC6915702; doi:10.1038/s41467-019-13761-7)
Supplement: Supplementary file 1 — Supplementary Information [file 41467_2019_13761_MOESM1_ESM.pdf]

Supplementary Note 1. Since the animals had free gaze, after some saccades during the search a target located in the affected, contralateral hemifield at the beginning of a trial ended up in the ipsilateral hemifield relative to fixation after those saccades, and conversely, a target located in the unaffected, ipsilateral hemifield at the beginning of a trial sometimes ended up in the contralateral hemifield relative to fixation after some saccades. During VPA inactivation sessions, such mismatches occurred with 18.8% and 18.9% of fixations in monkeys F and J, respectively. This switch of target location between hemifields could result in underestimating the behavioral effect of inactivation.

Supplementary Note 2. Consistent with our previous findings (Bichot et al., 2015), post-hoc tests found VPA inactivation increased the number of saccades to find the target from 4.7 to 5.7 in monkey F and 4.9 to 5.7 in monkey J (T-test; $t_{10} = 2.6$ and $t_{12} = 2.5$, $P = 0.025$ and $= 0.027$, for monkeys F and J, respectively), the total time to find the target ($t_{10} = 3.0$ and $t_{12} = 7.0$, $P = 0.013$ and $P < 10^{-4}$), as well as the error rates ($t_{10} = 3.6$ and $t_{12} = 3.9$, $P = 0.005$ and $P = 0.002$) when the target was in the contralateral hemifield. For monkey F, there was also a concomitant decrease in the total time to find the target ($t_{10} = 2.5$, $P = 0.031$) and error rates ($t_{10} = 2.9$, $P = 0.016$) when the target was in the ipsilateral hemifield. Effects of VPA inactivation on saccade latencies were different for the two monkeys, with no effects in monkey F (mixed two-way ANOVA, interaction factor; $F_{1,10} = 0.003$, $P = 0.96$) while, for monkey J, latencies increased for all target locations (post-hoc T-test; $t_{12} = 7.1$ and 10.2, $P < 10^{-4}$ and $P < 10^{-6}$, for ipsilateral and contralateral targets, respectively), but more so for contralateral targets (mixed two-way ANOVA, interaction factor; $F_{1,12} = 9.9$, $P = 0.008$).

Supplementary Note 3. Overall, in VPA inactivation sessions compared to control sessions, there was a decrease of 8.5% in saccades in the contralateral direction for monkey F (52.8% vs. 44.3%; T-test, $t_{10} = 6.2$, $P < 10^{-4}$) and 8.8% for monkey J (47.2% vs. 38.4%; T-test, $t_{12} = 3.6$, $P = 0.004$). This effect was present and significant for the first saccade after array onset as well as subsequent saccades for both monkeys, but it was more prominent for the first saccade compared to the subsequent ones (-24.8% vs. -4.9% and -17.3 vs. -6.2% for monkeys F and J, respectively). Eye movements in the vertical direction were negligible and amounted to less than 1% of all saccades regardless of monkey or session type). As a result of this selection bias in favor of ipsilateral stimuli, the total fraction of time spent searching the unaffected ipsilateral hemifield (relative to initial fixation) during inactivation sessions increased by 7.3% and 6.9% compared to control sessions in monkeys F and J, respectively. However, VPA inactivation did not significantly affect monkeys' tendency to look at stimuli that shared target features in the affected hemifield relative to fixations throughout the search (T-test; $t_{10} = 1.5$ and $t_{12} = 0.9$, $P = 0.18$ and $.38$, for monkeys F and J, respectively).

Supplementary Note 4. We also investigated whether the selectivity of the neurons for the different colors and shapes affected the feature selection effects for the target in the RF. To quantify the visual selectivity of the neurons throughout the session, we measured average responses to no-share distractors in the period of 70-135 ms after array onset during which neurons exhibit a robust evoked response that is not yet modulated by attention (Fig. 3A), and is independent of the period in which attentional modulation was measured (i.e., from 150 ms onwards). In both monkeys, neurons exhibited similar visual selectivity for colors and shapes, calculated as the ratio of the response to the most preferred color/shape and the response to the least preferred color/shape, in control vs. inactivation sessions (monkey F, color: control = 1.73, inactivation = 1.69, T-test $t_{93} = 0.3$, $P = 0.74$, shape: control = 1.34, inactivation = 1.33, $t_{103} = 0.5$, $P = 0.65$ - monkey J, color: control = 1.51, inactivation = 1.53, T-test $_{t141} = 1.1$, $P = 0.25$; shape: control = 1.20, inactivation = 1.17, $t_{128} = 1.2$, $P = 0.23$), with better selectivity for color than shape (T-test, monkey F, control: $t_{102} = 4.6$, $P < 10^{-4}$, inactivation: $t_{94} = 4.3$, $P < 10^{-4}$ - monkey J, control: $t_{123} = 11.6$, $P < 10^{-20}$, inactivation: $t_{146} = 18.5$, $P < 10^{-39}$). We then computed the correlation between the responses to the different colors and shapes in this period to the magnitude of attentional modulation (response to target – response to no-share distractor) during the analyses periods used previously when those colors and shapes were the target vs. when they formed a no-share distractor. We found no correlation between visual selectivity and feature-based attentional modulation in either monkey or session type before the first saccade ($P > 0.19$ for all comparisons) or subsequent saccades ($P > 0.23$ for all comparisons), with a general enhancement for the target across all features in control sessions, and no enhancement for the target across any feature in inactivation sessions. Results were the same when we limited the analysis to the most and least preferred colors and shapes. We compared the attentional modulation for the preferred features in the RF (i.e., preferred feature stimulus in RF and preferred feature search target – preferred feature stimulus in RF and non-preferred feature search target) to the attentional modulation for the non-preferred features in the RF (i.e., non-preferred feature stimulus in RF and non-preferred feature search target – non-preferred feature stimulus in RF and preferred feature search target) and found no significant difference in attentional modulation by preference before the first saccade (T-test; monkey F - control: $t_{51} = 0.3$, $P = 0.76$, inactivation: $t_{52} = 0.4$, $P = 0.70$; monkey J - control: $t_{82} = 0.6$, $P = 0.51$, inactivation: $t_{73} = 0.1$, $P = 0.90$) or before subsequent saccades (monkey F - control: $t_{51} = 1.2$, $P = 0.23$, inactivation: $t_{52} = 0.9$, $P = 0.39$; monkey J - control: $t_{82} = 1.7$, $P = 0.10$, inactivation: $t_{73} = 1.1$, $P = 0.27$). Previously we had shown that attentional effects are stronger for preferred features than non-preferred ones (Bichot et al., 2005), but the single-neurons recorded in that study were more strongly feature selective.

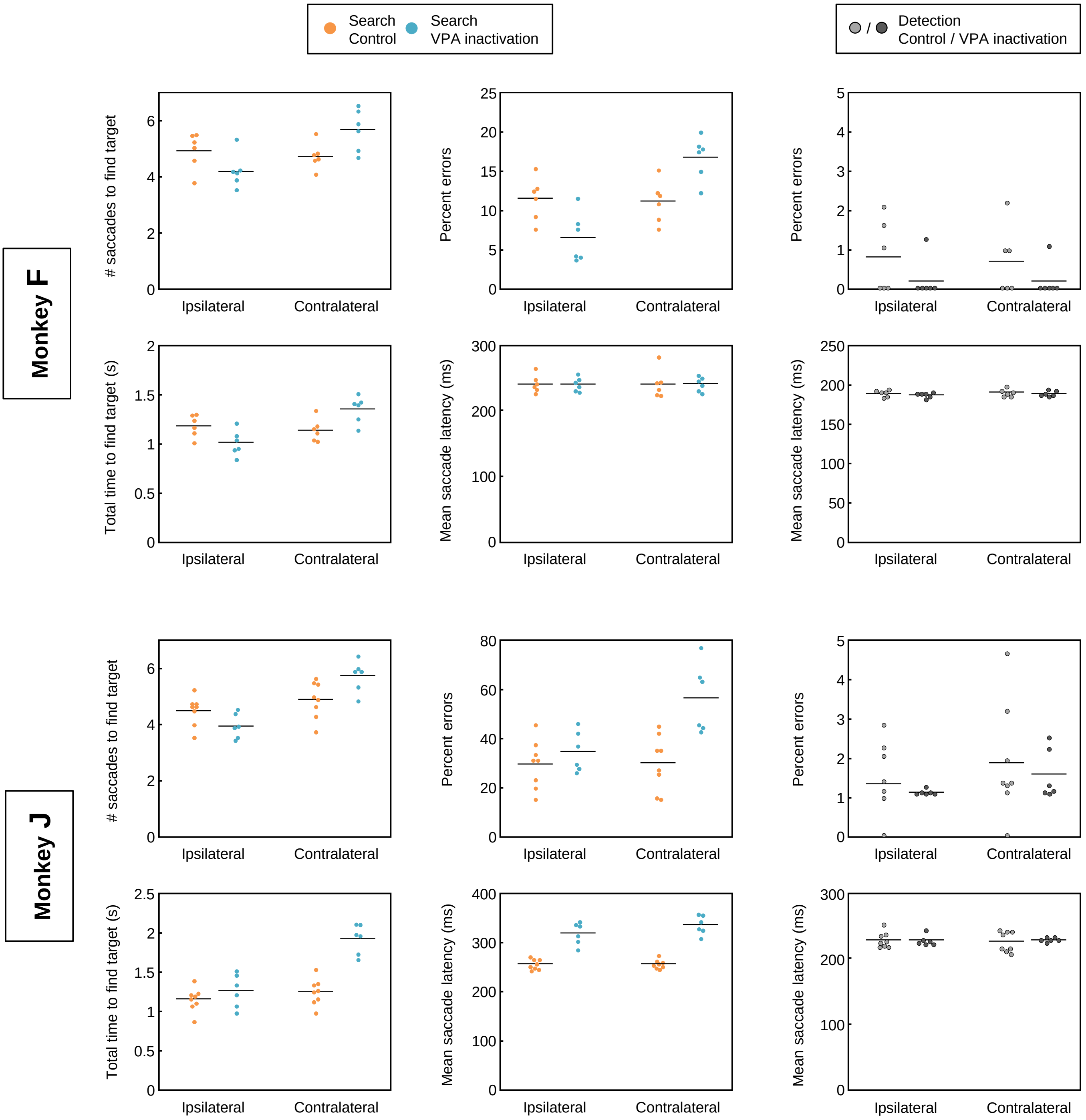

Supplementary Figure 1. Effect of VPA inactivation on behavioral performance. Individual session averages of the number of saccades to find the target, the total time to find the target, the error rate, and the saccade latency during control sessions (orange circles) and sessions in which VPA was inactivated (blue circles) are plotted as a function of search target location relative to the hemisphere in which VPA was inactivated. The averages of the number of saccades to find the target, the total time to find the target, and the saccade latency were calculated using correct trials only. Individual session averages of the error rate and the saccade latency during control sessions (light gray circles) and sessions in which VPA was inactivated (dark gray circles) are plotted as a function of detection target location relative to the hemisphere in which VPA was inactivated. Horizontal black lines represent across session averages which are shown in the bar plots of Figure 3.

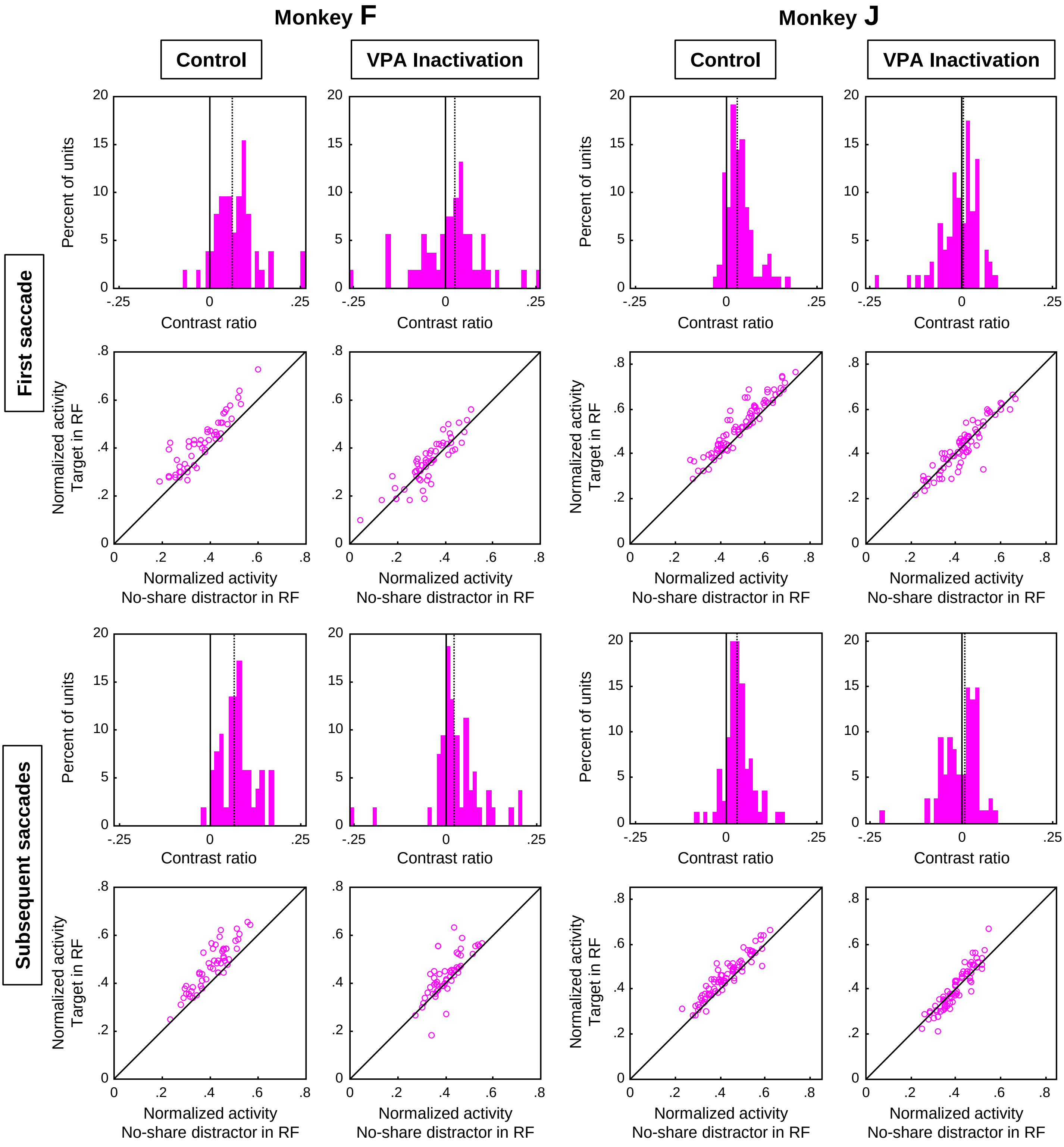

Supplementary Figure 2. Effect of VPA inactivation on feature selection in V4 during search. For each neuron, when a saccade was made to a stimulus outside the RF, the average response when the target was in the RF (i.e., red lines in Fig. 4) is plotted as a function of the average response when a no-share distractor was in the RF (i.e., blue lines in Fig. 4). The scatterplots are shown for control and VPA inactivation sessions for each monkey separately, and for activity before the first saccade and before subsequent saccades separately (i.e., middle vs. bottom row plots in Fig. 4). Contrast ratios were computed for each neuron as the difference in responses between the two attentional conditions of the scatterplots, divided by the sum of those responses. The barplots show the distribution of those contrast ratios. The leftmost possible bar (if present) represents all contrast ratios of less than -.25, and the rightmost possible bar (if present) represents all contrast ratios greater than .25. Dotted vertical lines mark the median contrast ratio.

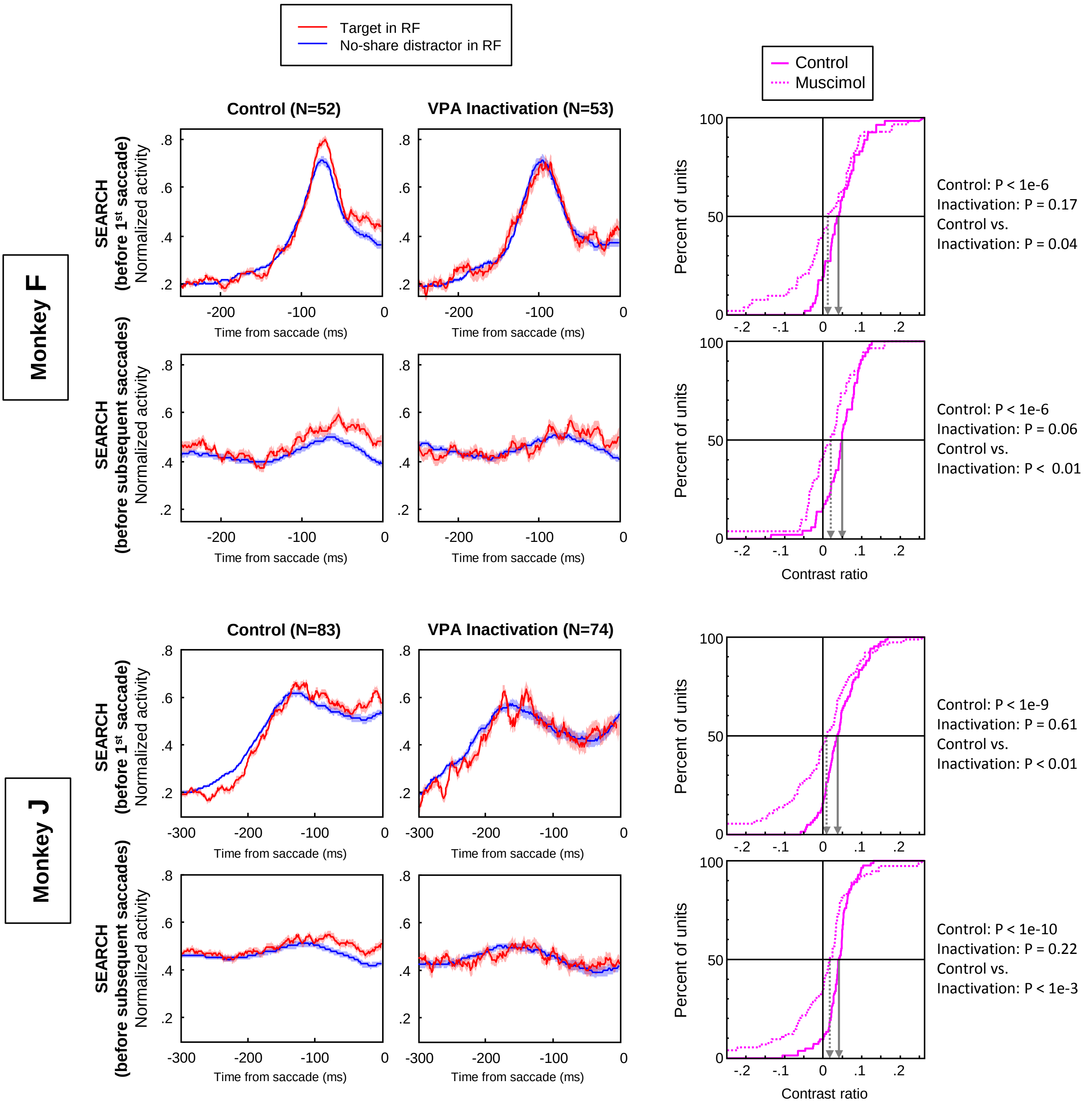

Supplementary Figure 3. Effect of VPA inactivation on feature selection in V4 during search with activity aligned on the time of saccade initiation. These plots show the attentional effects of Fig. 4 with activity aligned on the onset of the first saccade (top row) and the onset of subsequent saccades (bottom row) at time zero. For the top row, only spikes occurring after array onset were used, and for the bottom row, only spikes occurring after the end of the previous saccade were used in the calculation of neural responses. Cumulative distributions of attentional contrast ratios for control (solid magenta lines) and VPA inactivation (dotted magenta lines) sessions are also shown. Contrast ratios were computed for each neuron as the difference in responses between the two attentional conditions (i.e., target in RF vs. no-share distractor in RF), divided by the sum of those responses. The first point represents all contrast ratios of less than -.25, and the last point represents all contrast ratios greater than .25. The median contrast values for each session type is shown by the arrows (solid for control and dotted for Inactivation sessions). The significance value of the Wilcoxon signed rank test comparing the median of each distribution against zero, as well as of the Wilcoxon rank sum test comparing the mean contrast ratio between control and inactivation sessions is shown to the right of each cumulative distribution plot.

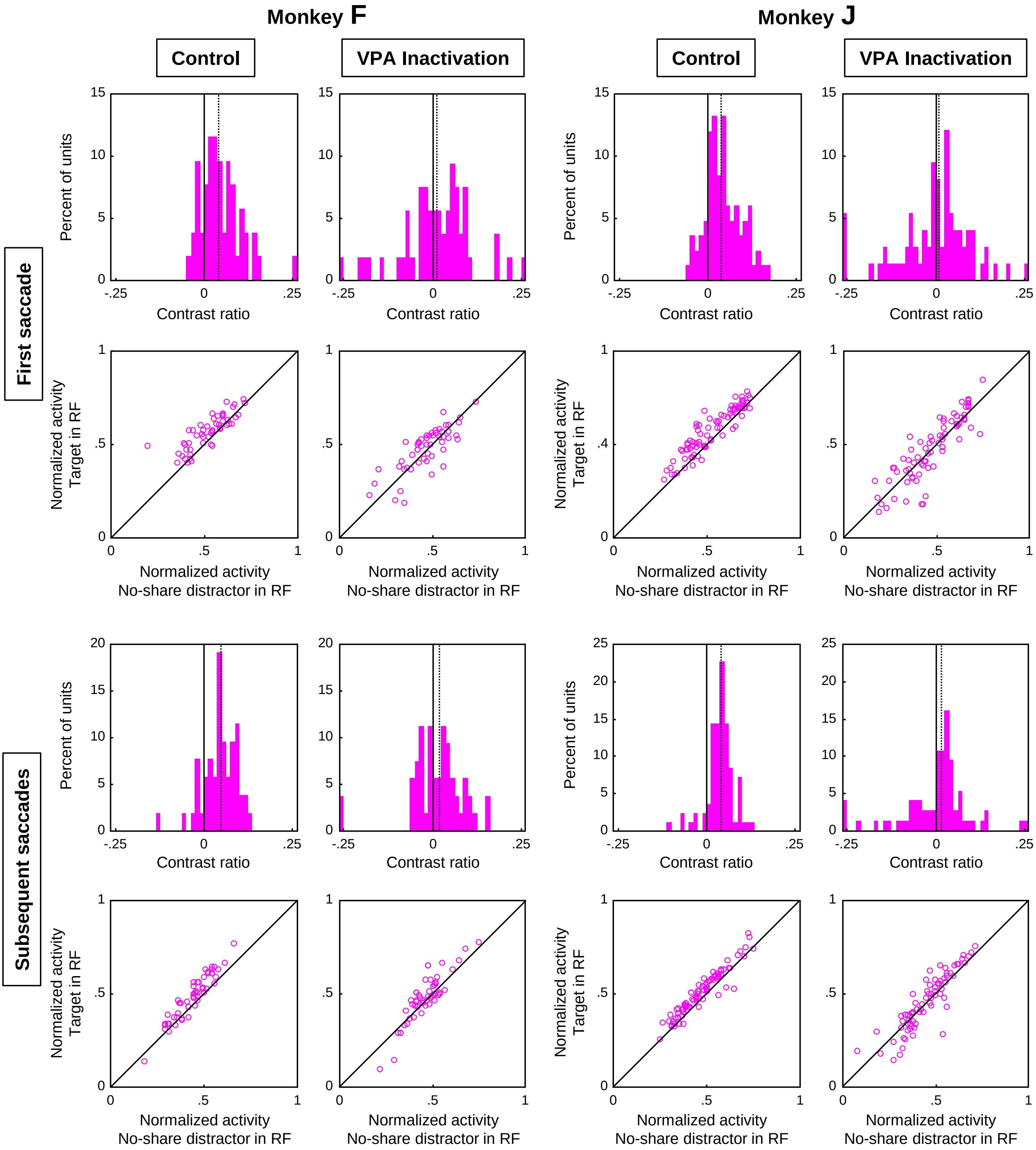

Supplementary Figure 4. Effect of VPA inactivation on feature selection in V4 during search with activity aligned on saccade initiation. Conventions as in Supplementary Figure 2.

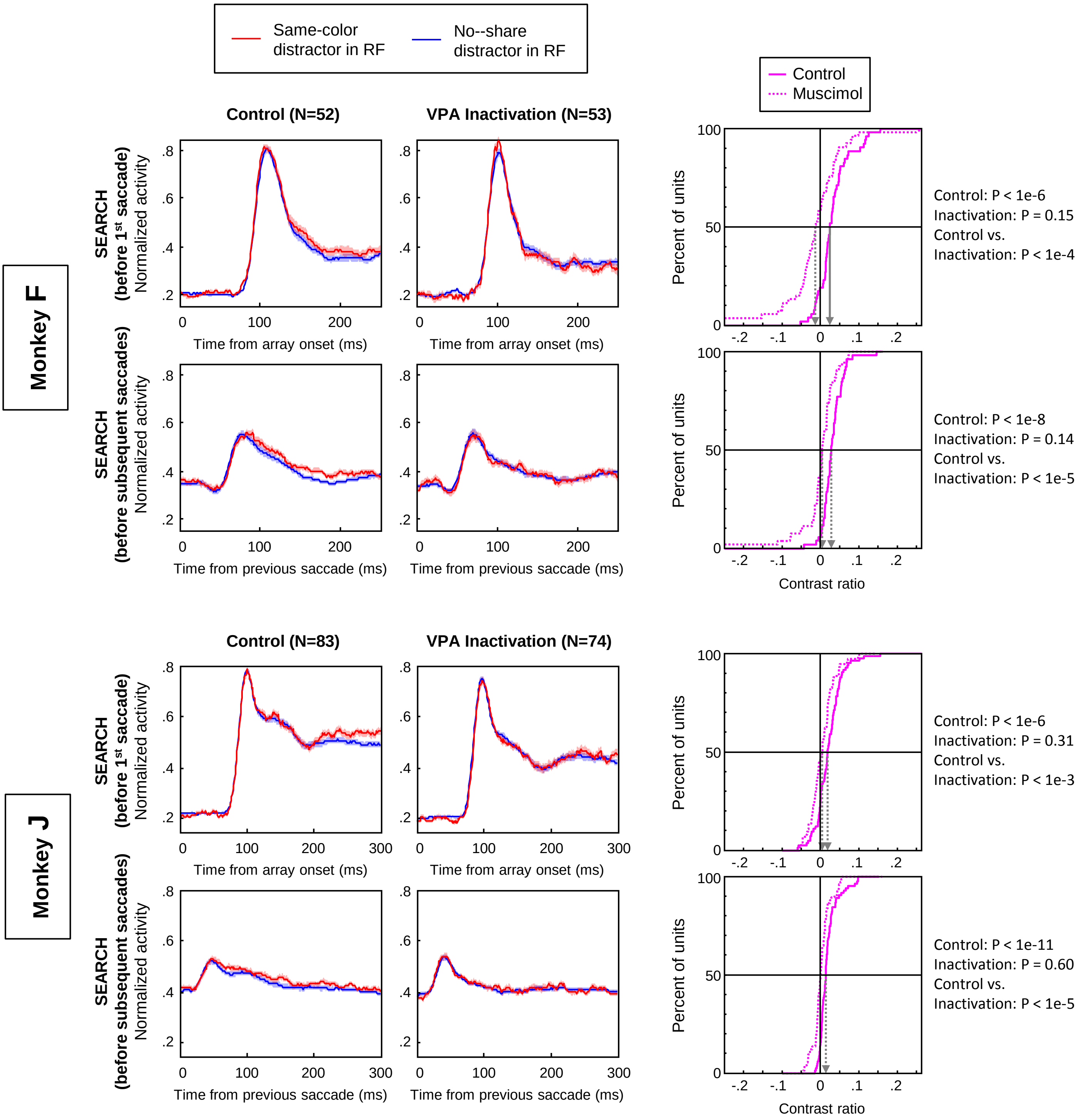

Supplementary Figure 5. Effect of VPA inactivation on color feature selection in V4 during search. We compared responses to a same-color distractor vs. a no-share distractor in the RF when a saccade was made away from the RF. All other conventions as in Fig. 4. We found a strong effect of VPA inactivation on selection before the first saccade (mixed two-way ANOVA; interaction of session type and stimulus type in RF, $F_{1,103} = 19.7$ and $F_{1,155} = 9.4$, $P < 10^{-4}$ and $P = 0.003$ for monkeys F and J, respectively) as well as before subsequent saccade ($F_{1,103} = 18.6$ and $F_{1,155} = 18.9$, $P < 10^{-4}$ for both). Follow-up post-hoc tests showed that while there were strong color selection effects during control sessions (T-test; before 1st saccade: $t_{51} = 5.3$ and $t_{82} = 5.8$, $P < 10^{-5}$ and $P < 10^{-6}$; before subsequent saccades: $t_{51} = 8.7$ and $t_{82} = 7.7$, $P < 10^{-10}$, for monkeys F and J, respectively), color selection effects in V4 were eliminated when VPA was inactivated (before 1st saccade: $t_{51} = 1.4$ and $t_{82} = 1.8$, $P = 0.16$ and $P = 0.08$; before subsequent saccades: $t_{51} = 0.2$ and $t_{82} = 1.0$, $P = 0.83$ and $P = 0.3$). The significance value of the Wilcoxon signed rank test comparing the median of each distribution against zero, as well as of the Wilcoxon rank sum test comparing the mean contrast ratio between control and inactivation sessions is shown to the right of each cumulative distribution plot.

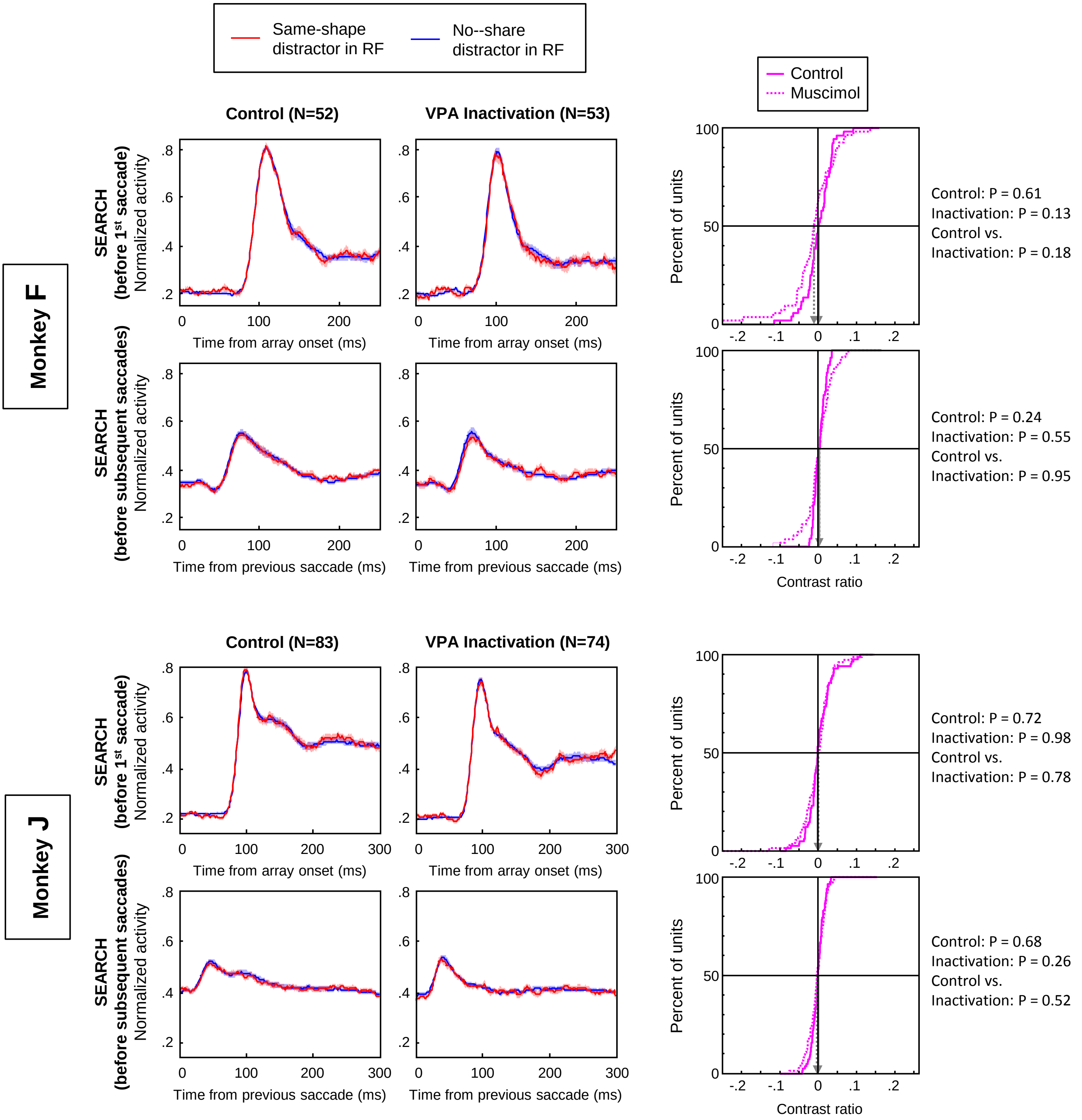

Supplementary Figure 6. Effect of VPA inactivation on shape feature selection in V4 during search. We compared responses to a same-shape distractor vs. a no-share distractor in the RF when a saccade was made away from the RF. All other conventions as in Fig. 4. Post-hoc tests showed that there were no shape selection effects during control sessions (T-test; before 1st saccade: t_{51} = 0.9 and t_{82} = 1.3, P = 0.38 and P = 0.21; before subsequent saccades: t_{51} = 1.8 and t_{82} = 0.4, P = 0.07 and P = 0.72, for monkeys F and J, respectively), or inactivation sessions (before 1st saccade: t_{51} = 0.8 and t_{82} < 0.01, P = 0.44 and P = 0.99; before subsequent saccades: t_{51} = 0.6 and t_{82} = 0.9, P = 0.52 and P = 0.38). Furthermore, there was no effect of session type on selection (mixed two-way ANOVA; interaction of session type and stimulus type in RF, before 1st saccade: $F_{1,103}$ = 1.3 and $F_{1,155}$ = 0.8, P = 0.26 and P = 0.38; before subsequent saccades: $F_{1,103}$ = 0.1 and $F_{1,155}$ = 0.2, P = 0.81 and P = 0.63, for monkeys F and J, respectively). The significance value of the Wilcoxon signed rank test comparing the median of each distribution against zero, as well as of the Wilcoxon rank sum test comparing the mean contrast ratio between control and inactivation sessions is shown to the right of each cumulative distribution plot.

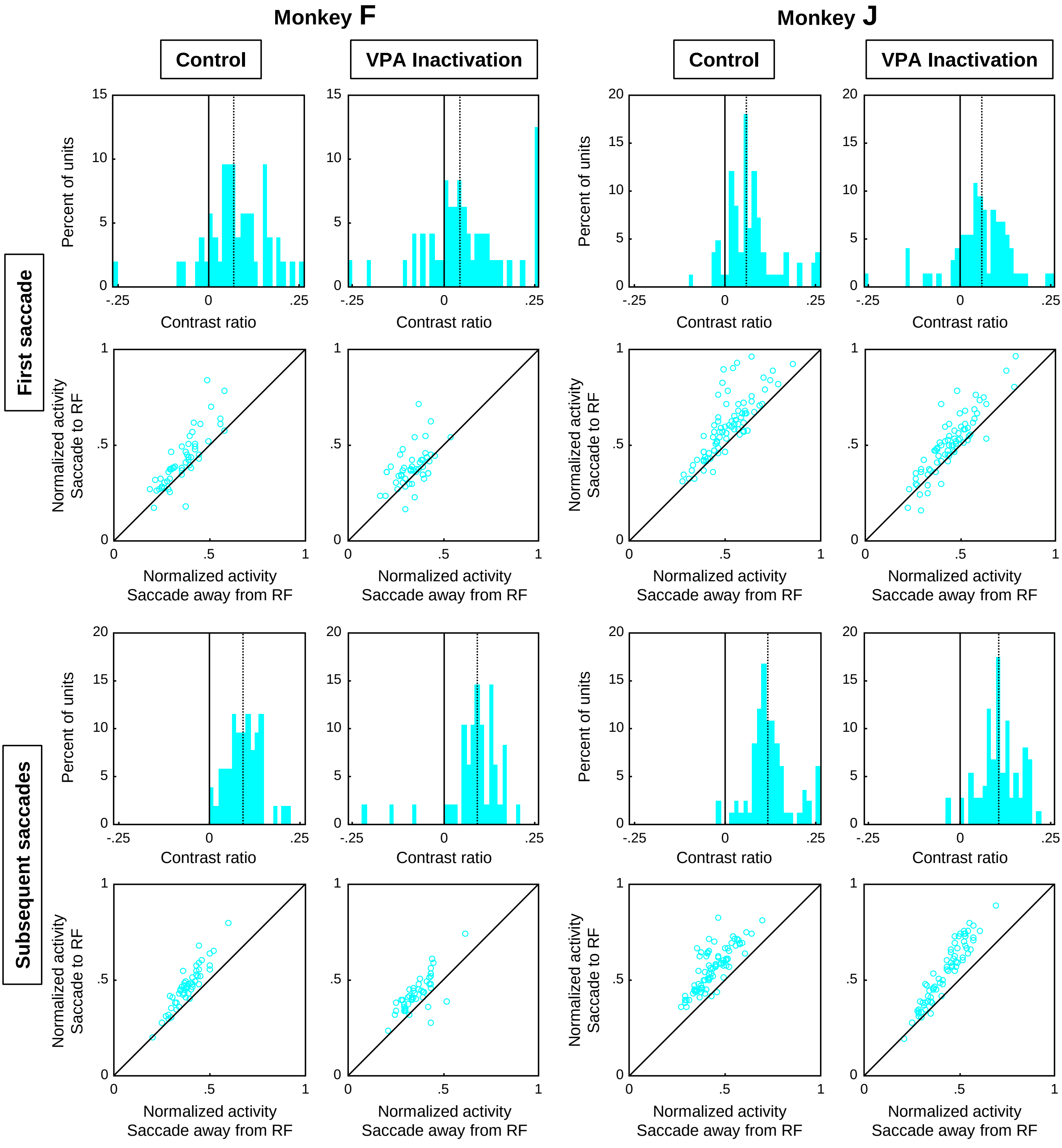

Supplementary Figure 7. Effect of VPA inactivation on spatial selection in V4 during search. For each neuron, the average response when a saccade was made to a stimulus in the RF (i.e., green lines in Fig. 6) is plotted as a function of the average response when a saccade was made to a stimulus outside the RF (i.e., blue lines in Fig. 6). The scatterplots are shown for control and VPA inactivation sessions for each monkey separately, and for activity before the first saccade and before subsequent saccades separately (i.e., top vs. bottom row plots in Fig. 6). Contrast ratios were computed for each neuron as the difference in responses between the two attentional conditions of the scatterplots, divided by the sum of the responses. The barplots show the distribution of those contrast ratios. The leftmost possible bar (if present) represents all contrast ratios of less than -.25, and the rightmost possible bar (if present) represents all contrast ratios greater than .25. Dotted vertical lines mark the median contrast ratio.
