# Supplementary material for: The role of prefrontal cortex in the control of feature attention in area V4
Source: Nat Commun. 2019 Dec 16;10:5727. doi: 10.1038/s41467-019-13761-7 (PMC6915702; doi:10.1038/s41467-019-13761-7)
Supplement: Supplementary file 3 — Reporting Summary [file 41467_2019_13761_MOESM3_ESM.pdf]

nature research

Corresponding author(s): Narcisse Bichot

Last updated by author(s): Nov 13, 2019

Reporting Summary

Nature Research wishes to improve the reproducibility of the work that we publish. This form provides structure for consistency and transparency in reporting. For further information on Nature Research policies, see Authors & Referees and the Editorial Policy Checklist .

Statistics

For all statistical analyses, confirm that the following items are present in the figure legend, table legend, main text, or Methods section.

n/a	Confirmed	
☐	☒	The exact sample size (n) for each experimental group/condition, given as a discrete number and unit of measurement
☐	☒	A statement on whether measurements were taken from distinct samples or whether the same sample was measured repeatedly
☐	☒	The statistical test(s) used AND whether they are one- or two-sided<br>*Only common tests should be described solely by name; describe more complex techniques in the Methods section.*
☒	☐	A description of all covariates tested
☐	☒	A description of any assumptions or corrections, such as tests of normality and adjustment for multiple comparisons
☐	☒	A full description of the statistical parameters including central tendency (e.g. means) or other basic estimates (e.g. regression coefficient) AND variation (e.g. standard deviation) or associated estimates of uncertainty (e.g. confidence intervals)
☐	☒	For null hypothesis testing, the test statistic (e.g. F, t, r) with confidence intervals, effect sizes, degrees of freedom and P value noted<br>*Give P values as exact values whenever suitable.*
☒	☐	For Bayesian analysis, information on the choice of priors and Markov chain Monte Carlo settings
☒	☐	For hierarchical and complex designs, identification of the appropriate level for tests and full reporting of outcomes
☐	☒	Estimates of effect sizes (e.g. Cohen's d, Pearson's r), indicating how they were calculated

Our web collection on statistics for biologists contains articles on many of the points above.

Software and code

Policy information about availability of computer code

Data collection	The experiments were under the control of a PC computer using MonkeyLogic software (University of Chicago, IL) that collected the behavioral data. Neural signals were amplified, band-pass filtered, digitized and collected using the Omniplex system (Plexon Inc.). Neural data was sorted offline using the Offline Sorter software (Plexon Inc.).
Data analysis	Data were analyzed using MATLAB (MathWorks, Natick, MA).

For manuscripts utilizing custom algorithms or software that are central to the research but not yet described in published literature, software must be made available to editors/reviewers. We strongly encourage code deposition in a community repository (e.g. GitHub). See the Nature Research guidelines for submitting code & software for further information.

Data

Policy information about availability of data

All manuscripts must include a data availability statement. This statement should provide the following information, where applicable:

- Accession codes, unique identifiers, or web links for publicly available datasets
- A list of figures that have associated raw data
- A description of any restrictions on data availability

The data and code that support the findings of this study are available from the corresponding author upon reasonable request.

Field-specific reporting

Please select the one below that is the best fit for your research. If you are not sure, read the appropriate sections before making your selection.

Life sciences study design

All studies must disclose on these points even when the disclosure is negative.

Sample size	The number of control and inactivation sessions were the same as in our previous study (Bichot et al., 2015) providing sufficient statistical power to conduct behavioral measure comparisons. We collected data from two monkeys (standard in behavioral neuroscience), and recorded from a total of 127 neurons in control sessions and 135 neurons in inactivation sessions with at least 50 neurons for each monkey and session type. The number of recorded neurons is well within accepted standards in the field.
Data exclusions	Only sessions in which monkeys correctly completed at least half of the trials of an experimental block (i.e., 1180/2=590) were used in the analyses.
Replication	Each recording session and each collected multi-unit neuronal data offers an independent data point. The behavioral and neural effects were confirmed in two monkeys with all analyses conducted separately for each monkey.
Randomization	The location and features (color and shape) of the target stimulus were randomized across trials for the visual search task. The location of the target was randomized across trials for the detection task. Visual search and detection trials were randomly interleaved.
Blinding	Not applicable.

Reporting for specific materials, systems and methods

We require information from authors about some types of materials, experimental systems and methods used in many studies. Here, indicate whether each material, system or method listed is relevant to your study. If you are not sure if a list item applies to your research, read the appropriate section before selecting a response.

Materials & experimental systems

n/a	Involved in the study	
x		Antibodies
x		Eukaryotic cell lines
x		Palaeontology
	x	Animals and other organisms
x		Human research participants
x		Clinical data

Methods

n/a	Involved in the study	
x		ChIP-seq
x		Flow cytometry
x		MRI-based neuroimaging

Animals and other organisms

Policy information about studies involving animals; ARRIVE guidelines recommended for reporting animal research

Laboratory animals	Two adult male rhesus macaque monkeys weighing 8-13 kg were used.
Wild animals	*Provide details on animals observed in or captured in the field; report species, sex and age where possible. Describe how animals were caught and transported and what happened to captive animals after the study (if killed, explain why and describe method; if released, say where and when) OR state that the study did not involve wild animals.*
Field-collected samples	*For laboratory work with field-collected samples, describe all relevant parameters such as housing, maintenance, temperature, photoperiod and end-of-experiment protocol OR state that the study did not involve samples collected from the field.*
Ethics oversight	The animals were cared for in accordance with the National Institutes of Health Guide for the Care and Use of Laboratory Animals and the guidelines of the MIT Animal Care and Use Committee.

Note that full information on the approval of the study protocol must also be provided in the manuscript.

October 2018
